# Methane Seep in Shallow-Water Permeable Sediment Harbors High Diversity of Anaerobic Methanotrophic Communities, Elba, Italy

**DOI:** 10.3389/fmicb.2016.00374

**Published:** 2016-03-31

**Authors:** S. Emil Ruff, Hanna Kuhfuss, Gunter Wegener, Christian Lott, Alban Ramette, Johanna Wiedling, Katrin Knittel, Miriam Weber

**Affiliations:** ^1^Department for Molecular Ecology, Max Planck Institute for Marine MicrobiologyBremen, Germany; ^2^HGF MPG Group for Deep Sea Ecology and Technology, Max Planck Institute for Marine MicrobiologyBremen, Germany; ^3^MARUM Center for Marine Environmental Sciences, University of BremenBremen, Germany; ^4^HYDRA Institute for Marine Sciences, Elba Field StationCampo nell’Elba, Italy; ^5^Department of Biogeochemistry, Max Planck Institute for Marine MicrobiologyBremen, Germany

**Keywords:** anaerobic oxidation of methane, sulfate-methane transition zone, ANME, microbial syntrophy, habitat heterogeneity, environmental selection, advection-driven ecosystem, Mediterranean

## Abstract

The anaerobic oxidation of methane (AOM) is a key biogeochemical process regulating methane emission from marine sediments into the hydrosphere. AOM is largely mediated by consortia of anaerobic methanotrophic archaea (ANME) and sulfate-reducing bacteria (SRB), and has mainly been investigated in deep-sea sediments. Here we studied methane seepage at four spots located at 12 m water depth in coastal, organic carbon depleted permeable sands off the Island of Elba (Italy). We combined biogeochemical measurements, sequencing-based community analyses and *in situ* hybridization to investigate the microbial communities of this environment. Increased alkalinity, formation of free sulfide and nearly stoichiometric methane oxidation and sulfate reduction rates up to 200 nmol g^-1^ day^-1^ indicated the predominance of sulfate-coupled AOM. With up to 40 cm thickness the zones of AOM activity were unusually large and occurred in deeper sediment horizons (20–50 cm below seafloor) as compared to diffusion-dominated deep-sea seeps, which is likely caused by advective flow of pore water due to the shallow water depth and permeability of the sands. Hydrodynamic forces also may be responsible for the substantial phylogenetic and unprecedented morphological diversity of AOM consortia inhabiting these sands, including the clades ANME-1a/b, ANME-2a/b/c, ANME-3, and their partner bacteria SEEP-SRB1a and SEEP-SRB2. High microbial dispersal, the availability of diverse energy sources and high habitat heterogeneity might explain that the emission spots shared few microbial taxa, despite their physical proximity. Although the biogeochemistry of this shallow methane seep was very different to that of deep-sea seeps, their key functional taxa were very closely related, which supports the global dispersal of key taxa and underlines strong selection by methane as the predominant energy source. Mesophilic, methane-fueled ecosystems in shallow-water permeable sediments may comprise distinct microbial habitats due to their unique biogeochemical and physical characteristics. To link AOM phylotypes with seep habitats and to enable future meta-analyses we thus propose that seep environment ontology needs to be further specified.

## Introduction

Methane seeps are widespread features of the seafloor along continental margins, where methane ascends from subsurface reservoirs and fuels methanotrophic communities or is emitted to the hydrosphere. The anaerobic oxidation of methane (AOM) is a key biogeochemical process regulating methane emission from marine sediments and is mediated by anaerobic methane-oxidizing archaea (ANME) and sulfate-reducing bacteria (SRB) (Knittel and Boetius, 2009). Marine AOM has mainly been investigated in deep-sea methane seeps, which are characterized by steep opposing gradients of methane and sulfate in the top centimeters of the sediment. In deep-sea cold seeps the sulfate-methane transition zones (SMTZ) have a thickness of only a few centimeters and are shaped by fluid flow and faunal activity (Bhatnagar et al., 2008; Fischer et al., 2012; Ruff et al., 2013; Felden et al., 2014). Between 20 and 80% of the methane (around 10^6^ tons of carbon per year) is consumed at the sediment water interface by methanotrophic microbial communities (Boetius and Wenzhöfer, 2013). Due to the aerobic water column the top layers of seep sediments are usually oxic and sustain aerobic methanotrophic bacteria, mainly of the gammaproteobacterial order *Methylococcales* (Lösekann et al., 2007; Tavormina et al., 2008; Wasmund et al., 2009; Ruff et al., 2013), whereas deeper sediment layers are depleted of oxygen and are dominated by AOM (Knittel and Boetius, 2009). Here, ANME and SRB usually form dense aggregates that occur at seeps in very high abundances resulting in cell numbers of 10^10^ cells per ml sediment at, e.g., Hydrate Ridge, the Black Sea (Knittel et al., 2005), Hikurangi Margin (Ruff et al., 2013), and Haakon Mosby mud volcano (Lösekann et al., 2007). Apart from methanotrophs and their partner bacteria, seeps comprise thiotrophic *Beggiatoaceae, Campylobacteraceae*, and *Helicobacteraceae* (Joye et al., 2004; Grünke et al., 2012; Felden et al., 2014) that often form thick mats on the seafloor. These organisms represent the methane seep microbiome, which is similar among deep-sea cold seeps worldwide, but very different from the surrounding seafloor (Ruff et al., 2015). The anaerobic organisms (ANME and their partner bacteria) are oxygen sensitive and it is yet unclear how they disperse between these isolated ecosystems, and whether coastal, dynamic sites harbor the same microbiome that establishes at deep-sea environments.

Shallow-water coastal methane seeps can be found at continental margins of all oceans, e.g., in the North Sea at 75–170 m water depth (Wegener et al., 2008), the East Timor Sea at 80 m (Wasmund et al., 2009; Brunskill et al., 2011), the Southeast Pacific at 1–5 m (Jessen et al., 2011) or the Northwest Atlantic at >50 m (Skarke et al., 2014). Coastal seeps at water depths of less than 100 m likely contribute large amounts of methane to the atmospheric budget as methanotrophs in the water column may oxidize only part of the emitted gas (McGinnis et al., 2006; Brunskill et al., 2011), e.g., a single shallow seep area off the coast of Chile emitted an estimated 800 tons of the potential greenhouse gas to the atmosphere per year (Jessen et al., 2011). Moreover, recent estimates indicate the presence of 1000s of coastal seeps worldwide (Skarke et al., 2014). However, despite their large number, their considerable methane emission, the biogeochemistry and microbial communities of coastal seeps are poorly understood.

The coastal seafloor is exposed to strong hydrodynamic forces caused by waves and tides. These high energies allow for the settlement of only larger particles of the sand fraction forming permeable sediments. Wave-driven advection furthermore greatly impacts the habitats of benthic microorganisms by the enhanced supply of electron donors, electron acceptors and nutrients (Precht and Huettel, 2004; Janssen et al., 2005), whereas deep-sea sediments in contrast are dominated by diffusive transport (Glud et al., 1994; Boetius and Wenzhöfer, 2013). Permeable coastal sediments harbor a high diversity of microorganisms (Mills et al., 2008) that are subjected to strong seasonal and spatial dynamics (Böer et al., 2009b; Gobet et al., 2012) due to changing abiotic conditions. It is yet unclear how these dynamics and the permeability of the sediment matrix influence the distribution, community structure, and activity of seep-associated microorganisms.

**FIGURE 1:**
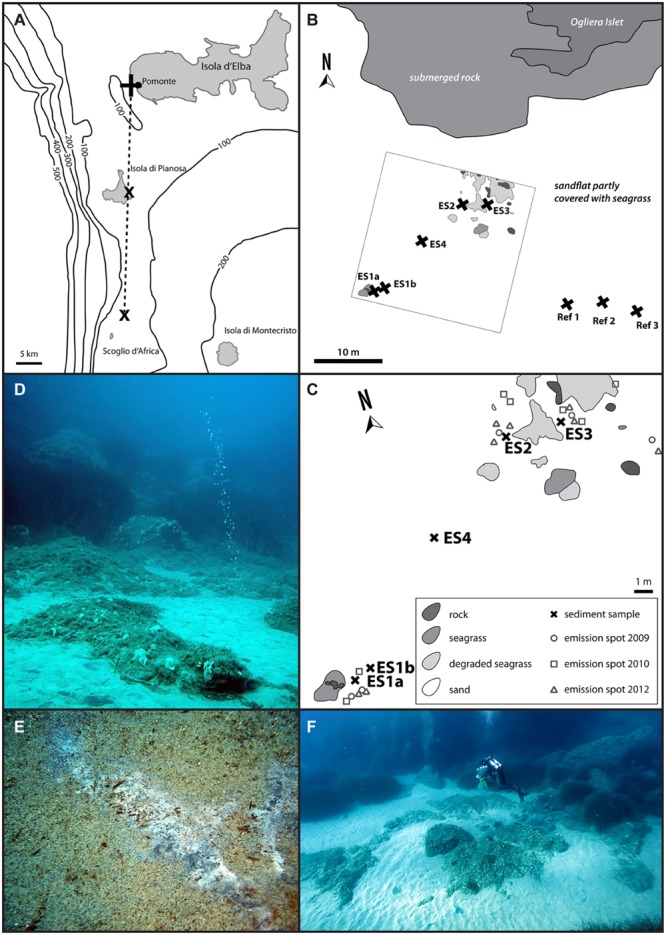
**Map of the Tuscan Island seep area **(A)** with the three major sites, close to the islands of Elba and Pianosa, and the islet Scoglio d’Africa.** This study focused on the Pomonte seep site **(B)** and nearby reference sites (Ref1–3). **(C)** Shows the detailed location of the investigated methane emission spots (ES1a, 1b, 2–4) at the Pomonte seep site. The emission spots are characterized by gas flares **(D)** as well as black sulfidic sediments that are occasionally covered by white mats of sulfur-oxidizing bacteria **(E)**. The emission spots are situated in 12 m water depth, surrounded by seagrass and rocks and are easily accessible by scuba diving **(F)**.

Here, we investigated shallow-water methane seepage off the coast of the Tuscan Island Elba (Italy). Elba is located in the Northern Tyrrhenian Sea, a relatively young (<15 Ma) back-arc basin formed by the roll-back of the Adriatic and Ionian subducting plates. The region is underlain by very thin continental crust and is tectonically very active (Greve et al., 2014 and references therein). Since 1995 the diving team of the HYDRA Field Station on Elba observe gas flares near the coast of the little village Pomonte, the island of Pianosa and the islet Scoglio d’Africa (**Figure 1**), which are all included in this tectonically active zone. At the Pomonte site the gas bubbles escape from permeable, organic carbon depleted sands and seagrass beds at around 12 m water depth. This unusual combination of tectonic setting, sediment characteristics and hydrodynamics turns the Pomonte seepage site into an outstanding ecosystem that differs from other known seep sites.

We focused this first investigation of the site on the detailed analysis of the biogeochemistry and microbial community structure. We chose four methane emission spots situated in these permeable sands and performed biogeochemical measurements, 16S rRNA gene sequencing and whole cell hybridizations. The study was based on three hypotheses: Methane seeps located in shallow, permeable sands (i) have characteristic biogeochemical profiles that are shaped by the profound hydrodynamic forces, (ii) harbor similar anaerobic methanotrophic communities than seeps found in the deep sea due to the strong selective pressure of methane as the predominant energy source, and (iii) have a higher diversity than deep-sea seeps, due to the greater number of niches available in coastal sands.

## Materials and Methods

### Site Description and Sampling Procedure

The investigated Pomonte methane seep site is part of the larger Tuscan Island seep area that is situated between the islands of Elba and Montecristo (**Figure 1A**). The Pomonte seep site (42°44.628′ N, 10°07.094′ E) is located 30 m off the Ogliera Islet at 12 m water depth (**Figure 1B**, Supplementary Table S1). The four investigated methane emission spots ES1–4 (**Figure 1C**) were situated in sandy patches between seagrass beds, showed bubble streams (**Figure 1D**) and lacked specific macrofaunal assemblages. The total investigated seep site had a size of 400 m^2^ and the individual emission spots (ES) were 5–10 m apart. We sampled ES1a and ES2 in 2009, ES1b, ES3, and ES4 in 2010, and in 2012 we sampled reference sediments that were located about 30 m away from the seepage site (Ref1–3). Sediment samples were taken with pushcores by scuba divers and were sectioned in 2 and 10 cm depth intervals quickly after retrieval. Subsamples of each section were carefully mixed and fixed overnight at 4°C in 2% formaldehyde in sterile artificial seawater, washed thrice with sterile seawater and stored in 1:1 seawater/ethanol at -20°C. The sediment for nucleic acid analyses was immediately stored in 70% ethanol at -20°C. 25 g sediment of selected samples was transferred to anoxic synthetic seawater medium [28 mM sulfate, 30 mM bicarbonate, pH 7.2, reduced with 0.5 mM disodium sulfide and supplemented with vitamins and trace elements according to Widdel and Bak (1992)] at room temperature for incubation and enrichment.

### Sediment and Pore Water Sampling and Analyses

The total organic carbon (TOC) content of the sediment was determined using a Carlo Erba NA-1500 CNS (Carbon, nitrogen, sulfur) analyzer with a precision of 0.2 wt% TC. Pore water was retrieved by *in situ* sampling using a 1 m long stainless steel pore water lance, and attached plastic syringes. One pore water profile was sampled at each emission spot ES1, ES2, ES3, and at one reference spot. Samples were taken every 10 cm, down to 60 cm maximum. For sulfide and sulfate measurements the samples were fixed under water with a 5% zinc acetate solution (final concentration = 45 mM), which was pre-filled into the syringes. The samples for DIC were filled headspace free into 2 ml boron silicate vials and fixed with 0.25 M mercury chloride (final concentration = 2.5 mM). For dissolved CH_4_ measurements, 16 ml porewater were added to 50 ml glass bottles containing 20 ml of 2.5% NaOH solution. The bottles were sealed with butyl rubber stoppers, vigorously shaken and stored upside down. All pore water samples were stored at 4°C. Sulfate concentrations were measured by non-suppressed anion exchange chromatography (Waters 430 Conductivity detector equipped with IC-Pak anion exchange column). Total dissolved sulfide concentrations were measured using the diamine complexation method (Cline, 1969). Concentrations of dissolved inorganic carbon (DIC) were determined by conductivity detection (Van Waters and Rogers Scientific, model 1054) using a flow injection setup (Hall and Aller, 1992) with 30 mM HCl and 10 mM NaOH as eluents, and isotopic compositions of inorganic carbon (δ^13^C_DIC_) by mass spectrometry (Finnigan MAT 252 connected to a gas bench and a GC-combustion system). Total alkalinity was measured by end-point titration (modified after Van Den Berg and Rogers, 1987) using 0.1 M HCl and a pH-meter with temperature probe (GPRT 1400 A, Greisinger electronic GmbH). Concentrations of dissolved methane were measured from alkalized headspace vials (2.5% NaOH) using a gas chromatograph (5890A Hewlett Packard) equipped with a Porapak-Q column (6 ft, 0.125 in, 80/100 mesh; Agilent, Santa Clara, CA, USA) and a flame ionization detector, operated at 40°C with helium as carrier gas. Technical replicates were not measured.

### Incubation Experiments

We performed radiotracer incubations at standardized conditions in artificial seawater medium with 28 mM sulfate (pH 7). Approximately 2 g of wet sediment was transferred into 5 ml Hungate tubes that were filled with medium as described above equilibrated with a 1.5 atmosphere CH_4_:CO_2_ (90:10) gas phase to study methane oxidation and methane-dependent (SR), or with a N_2_:CO_2_ gas phase (1.5 atm) to study methane-independent SR. AOM and SR rates were determined from replicate incubations (*n* = 5). To determine methane oxidation and SR we added 50 μl carrier-less ^14^C-methane tracer (15 kBq) or 20 μl carrier-less ^35^S-sulfate tracer (100 kBq) through a butyl rubber septum. Samples were incubated at room temperature for 3 days. Radiolabeled sulfate incubations were stopped by transferring the sample into 20% zinc acetate solution. We determined the activity of sulfate by directly transferring 100 μl sample into the scintillation cocktail (scintillation mixture; Ultima Gold, Perkin Elmer, Waltham, MA, USA; scintillation counter; 2900TR LSA; Packard Waltham, MA, USA). The production of radiolabeled inorganic sulfur was determined by cold chromium separation procedure (Kallmeyer et al., 2004) followed by scintillation counting as described above. SR rates were determined according to Jørgensen (1978). Radiocarbon incubations were stopped by sample transfer into gas-tight glass vials with 0.5 M NaOH solution. Methane concentrations were determined by gas chromatography from a headspace aliquot (Focus GC, Thermo equipped with a Poropak column; Analytical columns, Croydon, UK). The ^14^C-methane content was determined by gas phase stripping, combustion at 850°C with CuO and trapping in 2-phenylethylamine. ^14^C-inorganic carbon was released by acidification and trapped in 2-phenylethylamine (Treude et al., 2005). The activities of the fractions were measured as described above but using the scintillation cocktail Permafluor E+ (Perkin Elmer), and rates were determined as described before (Treude et al., 2005).

### Cultivation of Sediment Free AOM and Phototrophic Cultures

The AOM enrichment culture was started with sands from emission spot 1. The sands were diluted 1:1 with artificial seawater medium (Widdel and Bak, 1992) and incubated with methane as sole electron donor at 20°C in the dark. The concentration of sulfide was measured spectrophotometrically as colloidal copper sulfide that formed in an acidified copper sulfate solution (5 mM CuSO_4_; 50 mM HCl) according to Cord-Ruwisch (1985). In contrast to deep-sea sediments, which are silty and fine-grained, the microorganisms were embedded in silicate sands that quickly settled. Hence, we were able to collect the AOM organisms in the supernatant and easily remove the sandy matrix. The concentrated biomass (<1% of the total weight of the sample) regained around 60% of the microbial methane-dependent SR rate of the original sediment. The retrieved cultures needed to be stored in the dark as they also contained anaerobic phototrophs. We exchanged the medium when sulfide concentrations of >12 mM were reached, and we diluted AOM cultures (1:2) when the sulfide production in the enrichment vessel exceeded 0.25 mM per day. To enrich for the phototrophs we subjected a culture aliquot to sulfide (2 mM) and light. The rapidly growing phototrophs were further diluted (1:1000) and supplied with different sulfur sources (sulfide (2 mM), sodium sulfide (2 mM), or zero-valent sulfur).

### Nucleic Acid Extraction

Total nucleic acids were extracted from 2 ml sediment (in duplicates) using a chloroform-based method (Zhou et al., 1996) and from 1 ml sediment (in triplicates) using a Fast DNA Spin Kit For Soil (MP Biomedicals) according to the manufacturer’s recommendations. The nucleic acids were quality-checked by gel electrophoresis, concentration was measured by spectrophotometry (Qubit 2.0 Fluorometer and Infinite 200 Nano Quant) and the aliquots stored at -20°C until further processing.

### 16S rRNA Gene Libraries

We prepared 16S rRNA gene libraries from sediments of emission spot 1a (30–40 cm) and emission spot 3 (10–20 cm). Both sediment horizons showed the highest microbial activity and cell density of the respective seeps and thus were chosen to compare the most active seep communities. 16S rRNA genes were amplified by polymerase chain reaction (PCR) using ∼20 ng of environmental DNA, 30 cycles and the primer pairs GM3F/GM4R (Muyzer et al., 1995) for bacteria and Arch20F/Uni1392R (Lane et al., 1985; Massana et al., 1997) for archaea at annealing temperatures of 44 and 58°C, respectively. Ten replicate PCRs per sample were pooled, purified with the QIAquick Gel Extraction Kit (Qiagen) and eluted in ultrapure water. We cloned with the pGEM-T-Easy Vector System (Promega, Germany) and chemically competent *Escherichia coli* TOP 10 cells (Invitrogen, Germany). The inserts were amplified with the plasmid primer pair M13F/M13R (Yanisch-Perron et al., 1985), purified using Sephadex^TM^G-50 Superfine (GE Healthcare) and sequenced using the BigDye Terminator v3.1 Cycle Sequencing Kit (Applied Biosystems), the primers Bac907RM (Muyzer et al., 1998) for bacteria and Arch958R (DeLong, 1992) for archaea and an ABI Prism 3100 Genetic Analyzer or ABI 3130x1 Sequence Analyzer (Applied Biosystems). 16S rRNA gene sequences were quality-checked and assembled with Sequencher v4.6 (Gene Codes) and chimera checked with Mallard (v1.02) (Ashelford et al., 2006).

### Phylogenetic Analysis of 16S rRNA Gene Sequences

Taxonomic classification of the sequences was carried out using ARB (Ludwig et al., 2004) based on the SILVA small subunit rRNA reference sequence database base (SSURef v111, release date: 07–19–12) (Quast et al., 2013). Sequences were aligned with SINA and manually optimized based on their secondary structure. We calculated maximum likelihood (PhyML) trees based on 200 nearly full-length sequences (>1300 nucleotides) using 100 bootstraps and a positional variability filter, excluding highly variable positions. We added partial sequences (>590 nucleotides) to the tree using maximum parsimony as implemented in ARB, without allowing changes in the overall tree topology. Redundant sequences were removed for clarity.

### 16S rRNA Gene Pyrosequencing

Samples were amplified for pyrosequencing using forward and reverse fusion primers. The forward fusion primer was constructed with the Roche A linker (5′-CCATCTCATCCCTGCGTGTCTCCGACTCAG-3′), an 8–10 bp barcode, and the forward primer 340F (5′-CCCTAYGGGGYGCASCAG-3′) for archaea (Gantner et al., 2011), or the forward primer 341F (5′-CCTACGGGAGGCAGCAG-3′) for bacteria (Muyzer et al., 1993). The reverse fusion primer was constructed with a biotin molecule, the Roche B linker (5′-CCTATCCCCTGTGTGCCTTGGCAGTCTCAG-3′), and the reverse primer 958R (5′-YCCGGCGTTGAMTCCAATT-3′) for archaea (DeLong, 1992), or the reverse primer 907R (5′-CCGTCAATTCMTTTGAGTTT-3′) for bacteria (Muyzer et al., 1998). Amplifications were performed in 25 μl reactions with Qiagen HotStar Taq master mix (Qiagen Inc, Valencia, CA, USA), 1 μl of each 5 μM primer, and 1 μl of template. Reactions were performed on ABI Veriti thermocyclers (Applied Biosytems, Carlsbad, CA, USA) under the following thermal profile: 95°C for 5 min, then 35 cycles of 94°C for 30 s, 54°C for 40 s, 72°C for 1 min, followed by one cycle of 72°C for 10 min. Amplification products were visualized with eGels (Life Technologies, Grand Island, New York). Products were then pooled equimolar and each pool was cleaned and size selected using Agencourt AMPure XP (BeckmanCoulter, Indianapolis, Indiana) following Roche 454 protocols (454 Life Sciences, Branford, Connecticut). Size selected pools were then quantified and diluted to be used in emPCR reactions, which were subsequently enriched and sequenced following established manufacture protocols (454 Life Sciences). The PCR amplification of the 16S rRNA gene variable regions V3–V5, as well as amplicon purification, library preparation and massively parallel tag sequencing using a 454 GS FLX+ sequencer (Roche) was carried out at the Research and Testing Laboratory (Lubbock, Texas, USA). We processed the sequences using mothur v30 (Schloss, 2009) and a routine (Schloss et al., 2011) that included denoising of the flow grams (Quince et al., 2009), single-linkage preclustering (Huse et al., 2010) and the removal of chimeras (Edgar et al., 2011). Archaeal and bacterial sequences were clustered at 98% sequence identity (OTU_0.02_) and taxonomically assigned based on SILVA [release 119, 07-2014; (Quast et al., 2013)].

### Nucleotide Sequence Accession Numbers

16S rRNA partial gene sequences derived from Sanger-sequenced gene libraries were deposited under the accession numbers KT907894–KT908003. 16S rRNA amplicon sequences were deposited in the sequence read archive under SRA BioProject accession number SRP064784.

### Microbial Community Analyses

We used the original and subsampled sequence abundance tables to calculate diversity indices and Chao1 richness (Chao, 1984) using mothur v30. Dissimilarities between all samples were calculated using Bray–Curtis dissimilarity (i.e., relative sequence abundance) (Bray and Curtis, 1957). The resulting beta-diversity matrices were used for 2-dimensional non metric multidimensional scaling (NMDS) ordinations with 20 random starts (Kruskal, 1964). Stress values below 0.2 indicate that the multidimensional dataset is well represented by the 2D ordination. Taxa that were shared between sites or samples, as well as the networks were calculated using Jaccard dissimilarity (i.e., presences/absence). The network vertices (nodes) were plotted using a Fruchterman and Reingold (1991) force-directed algorithm, which causes an increase in the nodes attraction to each other with increasing similarity between them. For our dataset, it means that the more OTU_0.02_ two samples share, the closer they are located in the network. All analyses were carried out within the R software environment using the packages *vegan* (Oksanen et al., 2012), *labdsv* (Roberts, 2012), *gmt* (Magnusson, 2009), *network* (Butts, 2008), and custom R scripts.

### Catalyzed Reporter Deposition Fluorescence *In Situ* Hybridization

The sediment was sonicated (Sonoplus HD70 sonication probe, Bandelin, Berlin) seven times on ice (20 cycles, 30 s, 30% intensity). After each sonication step, 1 ml of supernatant was replaced with 1 ml 1:1 phosphate buffered saline (PBS)/ethanol and the supernatants combined. Depending on the sample we filtered 10–20 μl of supernatant onto a polycarbonate filter (0.2 μm pore size) and embedded the filter in 0.2% low-melting agarose to prevent detachment of cells. Filter sections were used for catalyzed reporter deposition fluorescence *in situ* hybridization (Pernthaler et al., 2002) as previously described (Ishii et al., 2004). For the detection of ANME-1 archaea (probe ANME1–350), cell walls were permeabilized with proteinase K solution (15 μg ml^-1^; Macherey–Nagel, 2.5 U mg^-1^) for 3 min at RT. For the detection of other archaea the cell walls were permeabilized with 0.5% SDS (Sigma) for 10 min at RT and bacterial cell walls were permeabilized with lysozyme solution (100kU ml^-1^; Sigma) for 45–60 min at 37°C. For hybridization we placed 4–6 filter sections in 500 μl hybridization buffer (150 ng ml^-1^ horseradish peroxidase-labeled oligonucleotide probe, see Supplementary Table S2) for 2 h at 46°C, incubated in washing buffer for 20 min at 48°C and in 1 × PBS for 15 min at RT. For signal amplification we placed the filter sections in 500 μl amplification buffer (1 mg ml^-1^tyramide) for 30 min at 46°C and washed twice with 1 × PBS for 10 min at RT. Cells were stained with DAPI solution (1 μg ml^-1^) and embedded in mounting medium (4:1 Citifluor/Vectashield). Details on buffers and solutions are also found on the web (ARB-SILVA Homepage: http://www.arb-silva.de/fish-probes/fish-protocols/). Cells were counted in 40–80 independent fields of view, corresponding to 600–2500 cells, using an Axioplan 2 mot plus epifluorescence microscope (Zeiss, Germany). Images were taken with a confocal laser-scanning microscope (LSM780 and LSM510; Zeiss, Germany) and processed with the software ZEN 2011 (Zeiss, Germany). Cell numbers in aggregates were estimated based on aggregate and cell volumes as previously described (Lösekann et al., 2007).

## Results

### Site Description and Ecosystem Conditions

The investigated Pomonte methane seep site is located 200 m off the coast of Elba (Italy) at 12 m water depth. Within the 400 m^2^ study area (**Figure 1C**) we observed seven emission spots with continuous methane bubble discharge (**Figure 1D**) of which four were analyzed in greater detail (**Figure 1C**, Supplementary Table S1). The location of the emission spots remained stable within half a square meter since the beginning of the investigation in 2009. All emission spots were characterized by permeable, silicate sands that were partly covered by degraded seagrass beds (**Figures 1D,F**). During calm weather conditions, usually in summer and winter season, the sediment at the spots was black and partly covered by white mats of filamentous bacteria (**Figure 1E**) that disappeared during storm events in autumn and spring. The sediment surface was repeatedly re-shaped by hydrodynamic forces to different ripple structures, indicating constant disturbance of the upper sediment layers. Despite these disturbances the surface at the emission spots ES2 and ES3 was black during most observation visits suggesting that the top sediment was anoxic/sulfidic. The surface of ES1 and ES4 was rarely colored black suggesting oxic top sediment. The sands recovered from the three seep sites had on average a grain size of 430 (± 34.6 SD) μm, a porosity of 43.2 (±0.96 SD) vol% and a permeability of 5.4 × 10^-11^ (±2.54 SD) m^2^. Reference sites had very similar characteristics [mean grain size: 448 (±38.8 SD) μm; mean porosity: 46.9 (±7.54 SD) vol%, mean permeability: 5.2 × 10^-11^ (±1.69 SD) m^2^], showing that seepage had a minor influence on the sediment characteristics. Total organic content of the sediment was very low at the emission and the reference spots [TOC 0.04 (±0.01 SD) wt%]. And inorganic carbon was 0.15 (±0.07 SD) wt% at the emission spots and 0.09 (±0.03 SD) wt% at the reference spots.

### Biogeochemical Characterization of the Emission Spots and Reference Sediments

**FIGURE 2:**
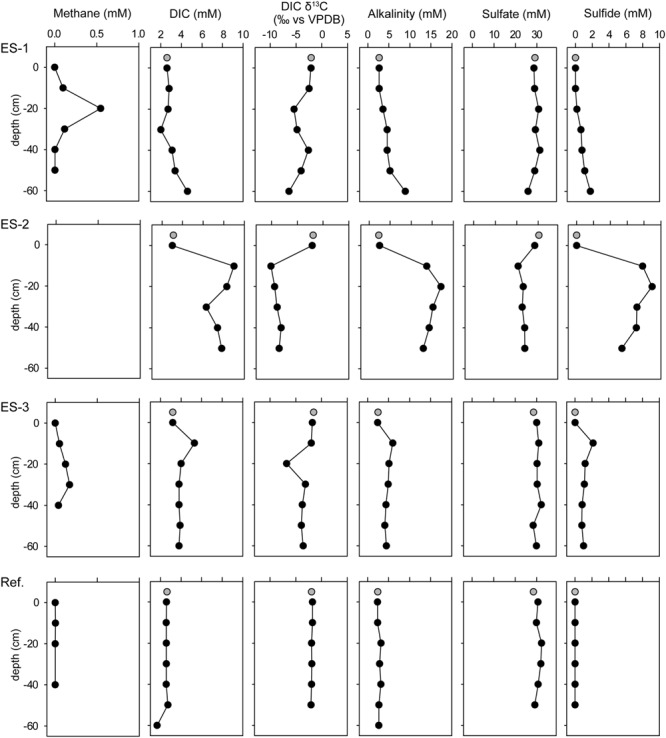
**Concentration profiles of methane, dissolved inorganic carbon (DIC), DIC isotopic signature (vs. VPDB), alkalinity, sulfate, and sulfide of the three investigated emission spots: ES1a, ES2, and ES3, as well as a reference spot (Ref).** Gray circles show water column measurements from 50 cm above seafloor.

The emitted gas contained up to 85% methane (and not further quantified proportions of ethane, propane, and CO_2_), with an unusual carbon isotopic signature of around ∂^13^C = -16aaaa vs. the Vienna Pee Dee Belemnite (VPDB) standard. This indicated abiogenic origin, which was further supported by the basement of this site consisting of fractured magmatic rock and the low organic carbon content of the sediment. We measured methane concentrations between 50 and 550 μM in the pore water (**Figure 2**), whereas in the reference sediments methane was below the detection limit (<1 μM; **Figure 2**). We assume, that locally the methane concentrations were rather underestimated, due to outgassing and constant dilution occurring in advective systems.

In the three investigated emission spots (ES1-3) the reactants and products of AOM were elevated. DIC, alkalinity and sulfide values, which also derived from *in situ* pore water sampling, were elevated compared to the reference sediments (**Figure 2**, Supplementary Table S3). Coinciding with the peaks of DIC, the isotopic compositions of inorganic carbon show values of down to δ^13^C_DIC_ = -10aaaa vs. VPDB. Notably in all three investigated spots, the sulfate concentrations were only slightly lower than in the overlying seawater and did not approach zero even at 50 cm sediment depth. Thus, AOM did not occur in classical, surficial SMTZ, but rather occurred vertically around the gas conduits to which sulfate is laterally supplied through the permeable sediment.

Anaerobic oxidation of methane and methane-dependent SR was measured in three horizons of emission spot 1 (Supplementary Figure S1). The highest methane oxidation (MOx) rates of 150 ± 50 nmol g^-1^ day^-1^ (±SD, *n* = 5) occurred in sediment depths between 20 and 60 cm and coincided with SR rates of up to 200 ± 40 nmol g^-1^ day^-1^ (*n* = 5). In controls without methane, SR rates were more than 10-fold lower, showing that microbial activity strongly depended on methane as energy source. In the upper 20 cm the rates of both MOx and SR were lower with 2 and 9 nmol g^-1^ day^-1^_._

### Preparation of Sediment-Free AOM Enrichment Cultures

To characterize the microbial processes involved in AOM it is desirable to have sediment-free microbial enrichments. Due to the slow growth of AOM-mediating organisms (Girguis et al., 2005; Nauhaus et al., 2007) it may take years of cultivation and sequential transfers to separate the cell material from the fine-grained sediments. The Pomonte seep sediments, however, consist of sands with very little organic carbon content. These sand grains settled very quickly in the medium. Thus, we were able to efficiently separate cell material from the sandy matrix within hours, retrieving about 60% of the AOM-active biomass in the original sediment. This enabled us to instantly obtain highly active, sediment-free enrichments with a methane-dependent sulfide production of about 0.3 μmol g_dryweight_^-1^ (see also Wegener et al., 2016). In a separate enrichment we found a phototrophic community of both green sulfur bacteria of the order *Chlorobiales*, as well as purple sulfur bacteria of the order *Chromatiales*.

### Microbial Diversity

**FIGURE 3:**
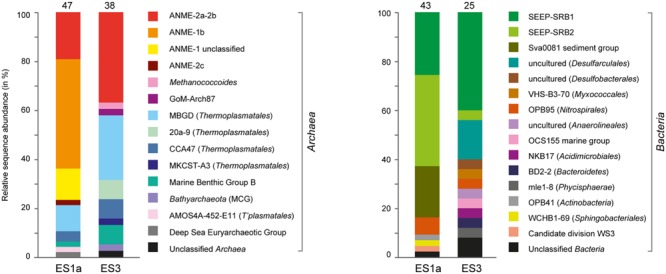
**Relative sequence abundances of archaeal and bacterial 16S rRNA genes at emission spot 1a (ES1a) and 3 (ES3).** The number of analyzed clones is given above the columns. The nomenclature of uncultivated groups is according to SILVA taxonomy.

**FIGURE 4:**
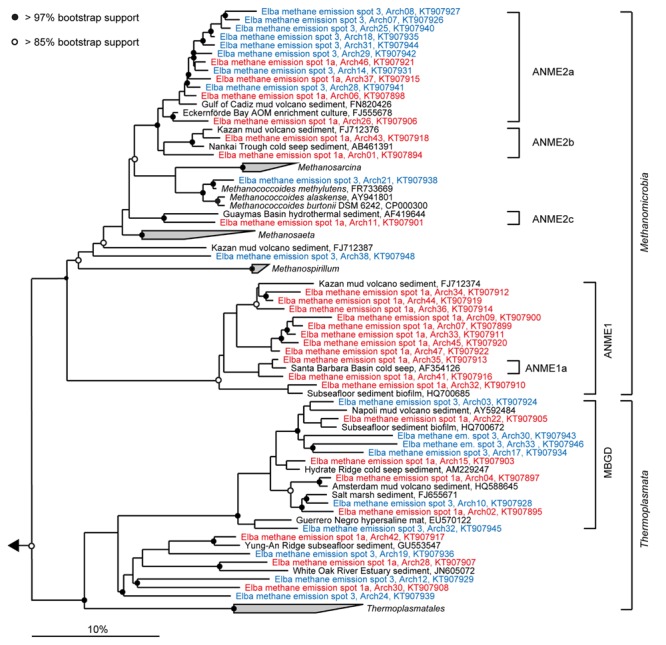
**Phylogeny of sequences affiliated with *Methanomicrobia* and *Thermoplasmata*.** The tree was calculated with nearly full-length 16S rRNA gene sequences (>1300 nucleotides) using maximum likelihood and 100 iterations. Partial gene sequences (>880 nt) retrieved from emission spots ES1a (red) and ES3 (blue) were added without changing the overall topology. The bar depicts estimated sequence divergence.

**FIGURE 5:**
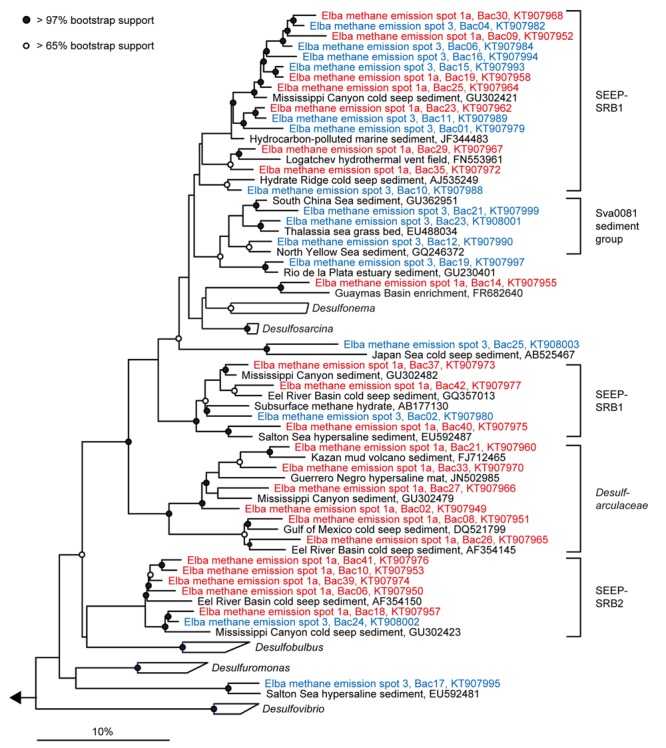
**Phylogeny of sequences affiliated with *Deltaproteobacteria*.** The tree was calculated with nearly full-length 16S rRNA gene sequences (>1300 nucleotides) using maximum likelihood and 100 iterations. Partial gene sequences (>590 nt) retrieved from emission spots ES1a (red) and ES3 (blue) were added without changing the overall topology. The bar depicts estimated sequence divergence.

**FIGURE 6:**
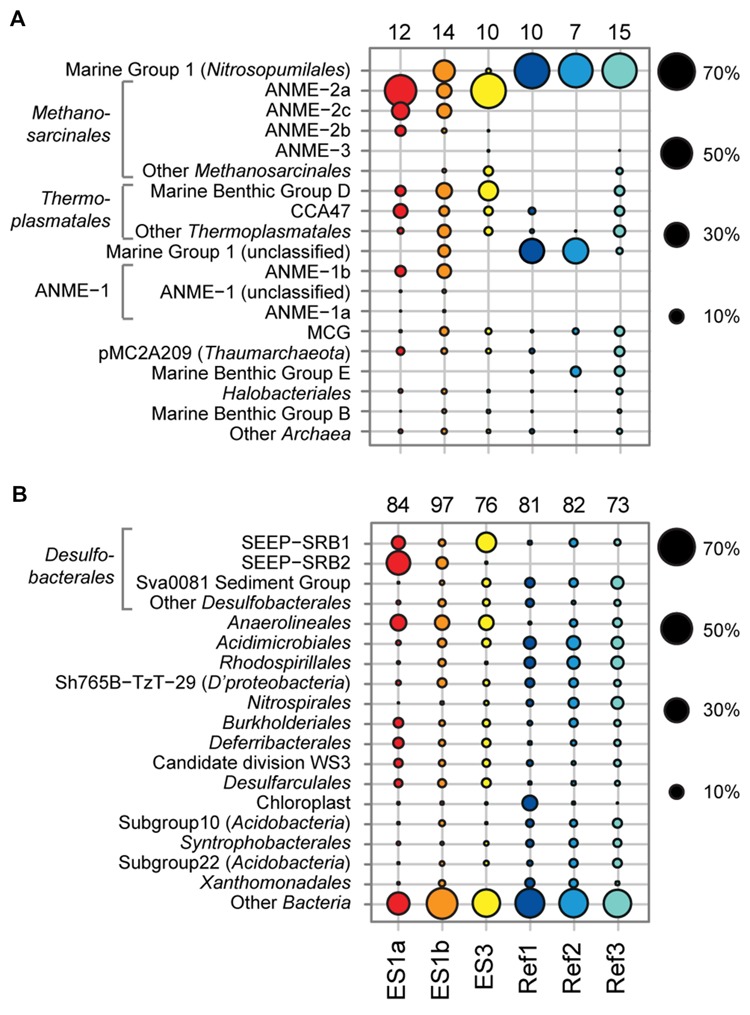
**Relative sequence abundances of archaeal **(A)** and bacterial **(B)** orders based on pyrosequencing of 16S rRNA genes (V3–V5 variable regions).** Archaeal and bacterial orders are sorted based on decreasing relative sequence abundance. The numbers above each column depict the total amount of order-level clades that were detected in the sample. *Methanosarcinales, Thermoplasmatales*, ANME-1, and *Desulfobacterales* are shown with major subclades.

The sediment horizons of ES1a (30–40 cm) and ES3 (10–20 cm) that showed the highest AOM activity were used for the construction of archaeal and bacterial 16S rRNA gene libraries. Despite the proximity and the observed geochemical similarity of the seep sites we found striking differences in their microbial richness and community composition (**Figure 3**). The libraries of ES1a were dominated by ANME-1 and SEEP-SRB2, and showed a high diversity of ANME clades, including ANME-2ab, ANME-2c, ANME-1a, and ANME-1b. The libraries of ES3 were dominated by ANME-2ab and SEEP-SRB1 and we did not detect any other ANME clades. In addition, only ES1a harbored sulfate reducers of the clade Sva0081, whereas only at ES3 we found *Desulfarculales*. The ANME and SRB at both seep sites were closely related to those found at other seeps worldwide (**Figures 4** and **5**). Pyrosequencing was performed with DNA from subsurface sediments of ES1a (30–40 cm), ES1b (20–30 cm), and ES3 (10–20 cm) to complement and extend the findings obtained by the gene libraries. As references we used subsurface sediments from three sites that showed no indication for methane seepage (Ref1–3). The numbers of observed archaeal and bacterial OTU_0.02_ (S) as well as estimated richness (Chao1) were similar at all emission and reference spots (**Table 1**), except for Ref2 that had a much lower S and Chao1. The highest archaeal and bacterial Inverse Simpson diversity (D) was found at ES1b, whereas ES3 showed an exceptionally low bacterial D compared to all other sediments. Despite their close proximity the emission spots harbored very distinct archaeal and bacterial communities and shared few OTU_0.02_. In general the turnover of OTU_0.02_ was lower within emission spots and within reference spots than between emission spots and reference spots (Supplementary Table S4), showing that emission spots were more similar to each other than the surrounding sediment. Eighty-three percent of all archaeal and 81% of all bacterial OTU_0.02_ occurred only at one location and we did not find one archaeal OTU_0.02_ that occurred in all six samples (Supplementary Figure S2). This high amount of uniqueness was found at emission spots and reference sites. The archaeal community at ES1a seemed to be dominated by ANME-2a, but also comprised ANME-2b, ANME-2c, ANME-1a, and ANME-1b (**Figure 6**). The bacterial community of the ES1a sample was dominated by SEEP-SRB2, but also contained SEEP-SRB1 and many *Anaerolineales*. ES1b was very similar to ES1a in its microbial composition. In contrast, the sample of ES3 was clearly dominated by ANME-2a, had sequences affiliated to ANME-3, but lacked ANME-2c and ANME-1. Here, the bacterial community was dominated by SEEP-SRB1.

**Table 1:** Diversity parameters based on pyrosequencing the V3–V5 region of sediment samples of the emission spots ES1a, ES1b, ES3, and the reference spots Ref1–3.

	Sample	Total reads	OTU_0.02_(S)^∗^	Chao1 richness (Chao1)^∗^	Inverse simpson diversity (D)^∗^
**Archaea**	ES 1a	14419	99	140	5.3
	ES 1b	3756	145	194	16
	ES 3	12103	255	463	3.1
	Ref 1	10462	150	270	5.6
	Ref 2	8601	40	64	3.4
	Ref 3	14296	257	428	6.0

**Bacteria**	ES 1a	3887	306	500	11
	ES 1b	4310	576	1057	202
	ES 3	6511	493	890	77
	Ref 1	6779	564	1166	177
	Ref 2	4254	469	766	141
	Ref 3	1444	558	1079	194

*OTU_*0.02*_: Operational taxonomic unit at 98% identity of the 16S rRNA gene region V3–V5.*

*^∗^Diversity parameters were calculated based on random subsampling to account for unequal sampling effort.*

### Relative Cell Abundance and Distribution of Microorganisms

**FIGURE 7:**
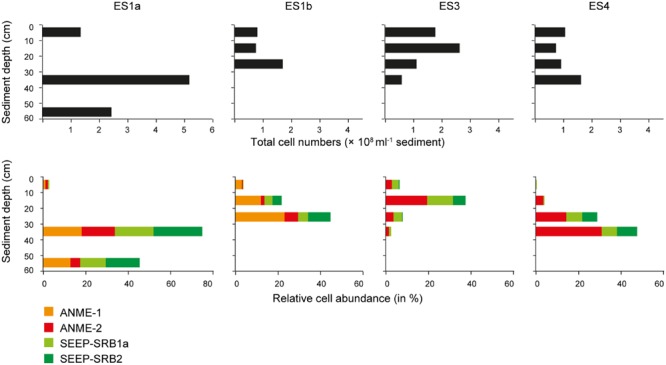
**Absolute cell abundance and relative cell abundances of keyplayers at the emission spots ES1a, ES1b, ES3, and ES4.** Absolute cell abundances **(upper panel)** were determined using Acridine Orange Direct Counts. Relative cell abundances of anaerobic methanotrophs and sulfate reducers **(lower panel)** determined by catalyzed reporter deposition fluorescence *in situ* hybridization (CARD–FISH).

**FIGURE 8:**
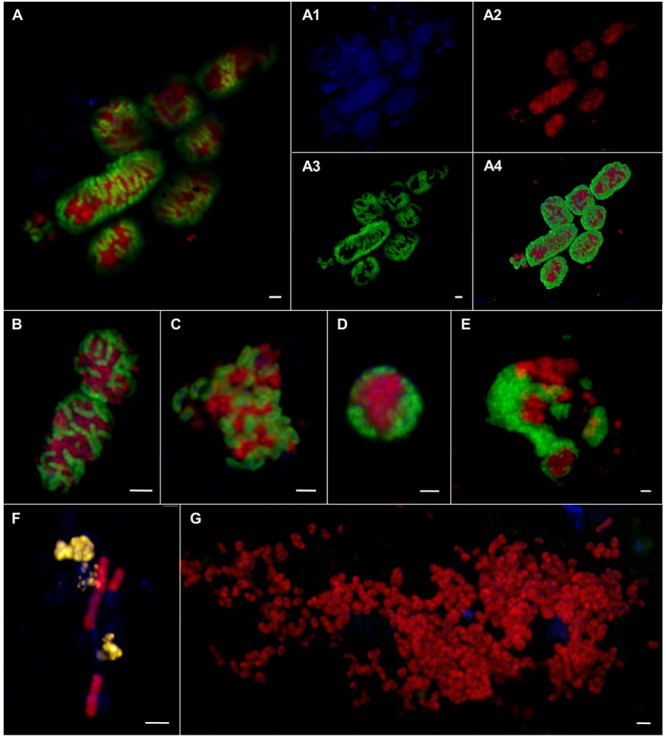
**Epifluorescence **(A–G)** and confocal laser scanning micrographs **(A1–A4)** of ANME and SRB associations visualized by CARD–FISH.** The woven-type consortia **(A,A1–A4,B)** consisted of ANME-1 or -2 (red, pooled probe: ANME-1/ANME-2) and SRB (green, probe: DSS658). Mixed-type **(C)**, shell-type **(D)**, and bubble-type **(E)** aggregates of ANME-2 (red, probe: ANME2-647) and SEEP-SRB1a (green, probe: SEEP1a-1441). Filamentous chain-type aggregate **(F)** of ANME-1 (red, probe: ANME1–350) and ANME-2 (yellow, probe: ANME2–538). Monospecific aggregate **(G)** of ANME-2 (red, probe: ANME2–538. Nucleic acids were stained with DAPI (blue). The scale bars are 2 μm.

The relative cell abundance of ANME-1, ANME-2, SEEP-SRB1a, and SEEP-SRB2 as determined by CARD–FISH varied substantially between seep sites and sediment layers. At all seeps the layer with the highest total cell abundance coincided with the highest relative abundance of anaerobic methanotrophs and sulfate reducers. These layers were between 10 and 40 cmbsf (cm below sea floor) and were highly dominated by ANME and SEEP-SRB (**Figure 7**). The CARD–FISH results confirmed that ES1a and ES1b were dominated by ANME-1 (∼20% of total cells; up to ∼9.5 × 10^7^ cells ml^-1^ sediment) and SEEP-SRB2 (∼25% of total cells). ES4 was dominated by ANME-2 (∼20% of total cells, up to ∼5 × 10^7^ cells ml^-1^ sediment) and SEEP-SRB1a (∼15% of total cells). ES4 had a similar community as ES3. At ES3 and ES4 we did not detect ANME-1 cells, indicating that this clade was entirely absent, as confirmed by both 16S rRNA gene libraries and pyrosequencing. Most of the ANME and SEEP-SRB formed conspicuous AOM consortia (**Figure 8**). Although we found all combinations, generally, ANME-1 was associated to SEEP-SRB2 and ANME-2 was associated to SEEP-SRB1a (**Figure 8**).

The morphology of these aggregates varied greatly ranging from consortia, in which ANME and SRB were interwoven (**Figures 8A,B**) to the typical mixed- and shell-type aggregates (**Figures 8C,D**) to aggregates that contained bubbles of cells (**Figure 8E**) or aggregates that formed long chain-like filaments (**Figure 8F**). Woven-type consortia had a compact cylindrical-shaped morphology with a densely packed archaeal core surrounded by SRB (**Figures 8A,B**). Here, the partner SRB enveloped the archaeal core without entirely covering it or growing into it, thus being a special case of the shell-type morphology. The archaeal core consisted very likely of ANME-1, since they were stained by an ANME-1/ANME-2 probe mix, but were not stained by ANME-2 probes. The envelope consisted of SEEP-SRB1a. Woven-type aggregations were on average between 12 and 36 μm long and between 4 and 8 μm thick. The bubble-type aggregates that we often observed were mat-like structures comprising mainly round-shaped or planar mono-species aggregates that are embedded in or next to each other (**Figure 8E**). These aggregates may also be considered special shell-type consortia. Bubble-type aggregates were very large being 15–75 μm wide and 6–10 μm thick. They contained ANME-1 or ANME-2 together with SEEP-SRB1a, SEEP-SRB2, and additionally cells of yet unknown clades that were only stained by DAPI. The SEEP-SRB1a mostly formed small, round and densely packed associations within the larger consortia, whereas SEEP-SRB2 regularly formed big and planar, but still tightly packed aggregates within the bubble-type consortium. SEEP-SRB2 was rarely detected in the usual shell-type aggregates. The chain-like aggregates were between 6 and 28 μm long and frequently consisted of a bundle of ANME-1 filaments and associated ANME-2, DSS, or SEEP-SRB2 cells.

**FIGURE 9:**
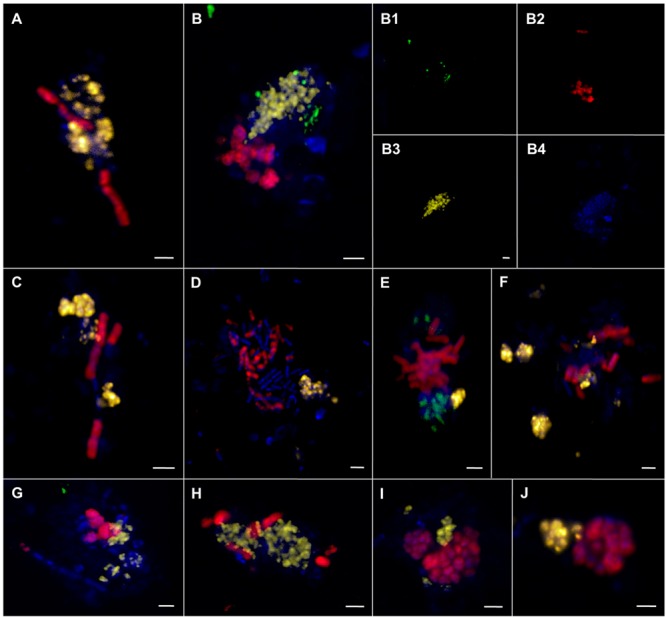
**Epifluorescence **(A–J)** and confocal laser scanning micrographs **(B1–B4)** of associations comprising ANME-1, ANME-2, and SRB visualized by CARD–FISH. (A–J)** ANME-1 (red) and ANME-2 (yellow) cells. **(B,E,G)** ANME-1 (red), ANME-2 (yellow), and SEEP-SRB1a (green) cells. DAPI stain in blue. The scale bar is 2 μm.

In sediments of ES1 we found the highest morphological diversity. The analysis of 366 aggregates of ES1, ES3 and ES4 revealed that filamentous chain-type aggregates were the most relative abundant type of consortia (18%), followed by bubble- and mixed-type (each 12%) and by shell-type aggregates (11%), whereas woven-type aggregates were rare (1%) (Supplementary Figure S3). Monospecific aggregates of ANME or SRB accounted for 29% of all aggregations. In addition, at ES1a and ES1b we found many aggregates that contained cells of both ANME-1 and ANME-2 (**Figures 8F** and **9**). These mixed ANME aggregates varied from loose associations to more tightly packed forms and were rather small being between 3 and 10 μm in diameter. The ANME-1 cells were mainly large and rod-shaped, while the ANME-2 cells were smaller and coccoid. ES3 and ES4 harbored microbial communities that were less phylogenetically and morphologically diverse. Here the community was dominated by shell-type aggregates and we did not detect any filament. At all emission spots we found monospecific aggregates of ANME or SEEP-SRB2. Monospecific ANME-2 aggregates (**Figure 8G**) had diameters from 6 μm to up to 90 μm and were frequently observed. ANME-1 monospecific aggregates were smaller (4–18 μm), less common and only occurred at ES1. Furthermore, we found ANME and SEEP-SRB single cells at all emission spots in low abundances. At the reference sites we did not detect any AOM consortia or monospecific ANME aggregates, but sporadically found single ANME or SEEP-SRB cells.

## Discussion

### Biogeochemistry of Shallow-Water Permeable Seep Sediments

Most of the so far studied methane seeps are located in muddy, silty deep-sea sediments that are less affected by hydrodynamic forces and temperature changes, and are thus very stable and permanently cold. The microbial communities at these deep-sea ecosystems develop over long periods of time and are predominantly shaped by faunal activity (Cordes et al., 2005; Thurber et al., 2012; Niemann et al., 2013; Ruff et al., 2013; Felden et al., 2014), or changes in the geochemistry (De Beer et al., 2006; Lichtschlag et al., 2010; Fischer et al., 2012; Felden et al., 2013; Zhang et al., 2014). The Tuscan Island seepage area harbors some of the shallowest marine methane seep sites investigated to date. The Pomonte seep site is outstanding as the emission spots are situated in permeable sands that are strongly influenced by diurnally and seasonally changing hydrodynamic forces such as waves and currents and by seasonal changes in water temperature from 12 to 25°C with an average temperature of 19.4 ± 4.3°C (Shaltout and Omstedt, 2014). In fine-grained or muddy seep sediments the transport of electron acceptors from the water column into the sediment is mainly diffusion regulated, or driven by small-scale advection due to gas ebullition, whereas in permeable sandy sediments this transport is largely due to advection-driven pore water circulation (Janssen et al., 2005). The pore water profiles indicate that advection and lateral transport of electron acceptors are important at all studied methane emission spots, which allow AOM activity in a deeper and wider sediment horizon than what is known from the deep sea. In the upper sediment horizons recurring disturbances such as sediment relocation or advective inflow of oxic water likely prevent settlement of the oxygen-sensitive AOM consortia.

In deep-sea methane seep sediments the sulfate-methane transition zone (SMTZ) and hence the zone of highest activity is usually only a few centimeters thick. Often the SMTZs are located close to the sediment surface and harbor between 10^9^ and 10^10^ microbial cells per milliliter sediment (Knittel and Boetius, 2009). At the Pomonte shallow seep site, the zone of highest AOM activity harbored 10^8^ microbial cells per milliliter sediment, was much deeper and between 20 and 40 cm thick. The sulfate concentration did not decrease as is the case in deep-sea sediments and seeps, but stayed fairly stable down to 60 cm sediment depth, indicating that sulfate replenishes from the surrounding sands. Thus, at the Pomonte site we do not find a classical SMTZ, with sharp vertical gradients of methane and sulfate, commonly found in surface sediments of methane seeps (e.g., Lichtschlag et al., 2010; Ruff et al., 2013), or in the subsurface of seeps and methane rich sediments (Webster et al., 2011; Treude et al., 2014). We instead observed that AOM activity mainly occurred in deep sediment layers and that AOM aggregates were distributed throughout the sediment cores. This is unusual for methane seeps and indicated that the active zone, including opposing gradients of methane, and sulfate, was found laterally around the central gas conduit, rather than being confined to a thin layer close to the sediment surface. This hypothesis is further supported by studies showing that the upward flow of gas creates a downward flow of seawater in adjacent sediments (O’Hara et al., 1995; Tryon et al., 1999), which may result in lateral advection of seawater through the permeable sediment around a central conduit (Stein and Fisher, 2001).

Both methane and sulfate occurred in excess throughout the sediment and were not depleted, indicating a low efficiency of the benthic filter in permeable, low-biomass sands. A large part of the methane that passes through the sand without being oxidized also passes through the shallow water column, making its way to the atmosphere, where it may act as a potent greenhouse gas (McGinnis et al., 2006; Brunskill et al., 2011). Methane seepage was shown to cause similar biogeochemical profiles in other mesophilic, permeable sandy sediments, e.g., at Coal Oil Point in the Santa Barbara Basin (Treude and Ziebis, 2010), the Skagerrak (Knab et al., 2008) and the Gulfaks Oil Field in the North Sea (Wegener et al., 2008). Highly permeable seep sediments also exist in hydrothermal settings, e.g., at Middle Valley on the Juan de Fuca Ridge (Wankel et al., 2012), showing similar lateral advection of overlying seawater (Stein and Fisher, 2001). Based on these observations it is possible that methane venting through permeable sands generally features characteristic biogeochemical profiles, vertical SMTZs and low-efficient biofilters.

Many studies in recent years have tried to elucidate the niche differentiation and ecophysiology of populations that are directly or indirectly involved in the anaerobic oxidation of methane and/or hydrocarbons. Although, evidence is accumulating that microbial populations differentiate based on the availability of electron acceptors (Green-Saxena et al., 2014), electron donors (Grünke et al., 2011), hydrocarbons (Stagars et al., 2016), temperature (Holler et al., 2011b), and the substratum (Case et al., 2015), to name just a few, the ecological processes and niches remain unclear. To link phylotypes with habitats it is necessary to define environment ontologies (Buttigieg et al., 2013; Thessen et al., 2015) that can be used to clearly distinguish different types of seep ecosystems and AOM habitats. The term “seep” that we used throughout this study is strictly speaking a misleading description for the investigated sites, as they have characteristic gas flares and are continuously shaped by gas ebullition.

### Diversity and Turnover of Microbial Communities in Shallow, Permeable Seep Sediments

Diversity and turnover of the microbial communities at the Pomonte site were described and compared to those found at deep-sea methane seeps. The microbial communities of Elba shallow seeps comprised organisms that were closely related to those found at other seep ecosystems worldwide (**Figures 4** and **5**). We detected 16S rRNA partial gene sequences that shared >97% sequence identity with 16S ribosomal genes of ANME or SRB organisms that occurred, e.g., at methane seeps in the Nankai Trough (Miyashita et al., 2009), the Santa Barbara Basin (Orphan et al., 2001), the Gulf of Mexico (Lloyd et al., 2010) at mud volcanoes in the Mediterranean (Pachiadaki et al., 2010), and the Atlantic (Niemann et al., 2006), and in SMTZs of organic-rich shallow sediments of Eckernförde Bay (Jagersma et al., 2009). Hence, the biogeochemical and physical constraints of the permeable microbial habitat selected for the same microbial communities that are involved in AOM worldwide. Yet, despite their proximity and their biogeochemical similarities, the studied emission spots were remarkably different concerning their richness and evenness based on amplicons of the V3–V5 region (**Table 1**) as well as concerning their dominant microbial clades based on both partial genes (**Figure 3**) and V3–V5 amplicons (**Figure 6**).

Permeable sands are much more heterogeneous than soft deep-sea sediments and provide a large number of niches to microorganisms (Mills et al., 2008; Böer et al., 2009a; Schöttner et al., 2011; Gobet et al., 2012). This heterogeneity may result in an increased number of niches also for anaerobic methanotrophs and sulfate-reducers. The high diversity of key players that we observed may be connected to frequent changes in the concentration of methane, sulfate and especially oxygen caused by lateral advection of seawater to the deeply buried, but permeable AOM zones. At the emission spots ES1a and ES1b we found ANME-1a, -1b, -2a, -2b, -2c and ANME-3 as well as SEEP-SRB-1a, -2, and many other sulfate reducers, among them Sva0081 and *Desulfarculales*. This indicated the coexistence of clades with very different habitat preferences, such as ANME-1, which predominantly occur in anoxic, deep (ANME-1b) or hot (ANME-1a) sediment layers (Biddle et al., 2012; Vigneron et al., 2013; Ruff et al., 2015), seem to be oxygen sensitive (Knittel et al., 2005) and tolerant to changes in temperature and sulfate availability (Dowell et al., 2016) and ANME-2c, which seem to be adapted to very different conditions like shallow, bioirrigated and sulfate-rich sediment layers (Biddle et al., 2012; Vigneron et al., 2013; Felden et al., 2014). Moreover, we observed associations that contained cells of both ANME-1 and ANME-2. To our knowledge this is the first time that such associations were visualized, despite the frequent co-occurrence of ANME clades in sequence-based studies of seep microbial diversity (e.g., Ruff et al., 2015). Monospecific occurrence of ANME archaea has been described before (Orphan et al., 2002; Lösekann et al., 2007; Treude et al., 2007; Wegener et al., 2008; Vigneron et al., 2013), but the causes remain elusive. Aggregation could be a strategy to minimize stress caused by advection-driven entrainment of oxygen, which was shown for sulfate reducers (Dolla et al., 2006). For sulfate-coupled AOM most physiological studies emanate from obligate syntrophy of ANME and SRB (Girguis et al., 2005; Nauhaus et al., 2005; Orphan et al., 2009; Meulepas et al., 2010; Holler et al., 2011a; McGlynn et al., 2015; Wegener et al., 2015) with findings that seemingly make monospecific life unfavorable. We also detected methanogens and sulfur disproportionating bacteria, which frequently occur at methane seeps and in AOM enrichment cultures. These organisms are very widespread, but rare and are involved in side reactions of AOM (Wegener et al., 2016). In addition, the phototrophic enrichments suggested that sulfide-oxidizing phototrophs inhabit the sediment surface of the seeps, using the AOM-derived sulfide as an electron donor for photosynthesis. This indicated that shallow seeps are a so far overlooked habitat for anoxygenic green and purple phototrophs.

Phylogenetic diversity was paralleled by an unprecedented morphological diversity, including several different forms of spherical and filamentous consortia, monospecific aggregates and even indications for AOM consortia that are comprised of two ANME clades and one SRB (**Figures 8** and **9**). It was shown that cell aggregation decreased both cell movement through a sand column (Vandevivere and Baveye, 1992) and the likelihood of being grazed by benthic predators (deLeo and Baveye, 1997), and may even enhance substrate uptake per cell (Logan and Hunt, 1987). In addition, grain size and permeability can influence both the abundance (Santmire and Leff, 2007) and the community structure of benthic communities (Jackson and Weeks, 2008; Zheng et al., 2014). It is possible that the high phylogenetic and morphological diversity is linked to the permeability or other parameters of the sediment. The different aggregate morphologies could be adaptations to different flow regimes, interstitial spaces, and compound concentrations. It was shown that disturbances, such as the exposition to oxygen (Shade et al., 2011) or salinity (Berga et al., 2012), influence community composition and function, while other disturbances may increase the microbial diversity of an ecosystem (Flöder and Sommer, 1999; Buckling et al., 2000). Yet, the effects of sediment characteristics and disturbance largely remain understudied in particular in ecosystems that are difficult to reach and sample, such as most methane seeps.

Disturbance caused by the strong hydrodynamics may also explain the high microbial turnover between the sites. Other factors may include the fluctuations in immigrating and emigrating microbial populations, which is a stochastic process that is especially important in dynamic habitats (Böer et al., 2009b; Gobet et al., 2012), or energy-diversity, as these sands are natural filters of fresh organic matter particles from both marine and terrestrial sources. It was shown that differences in available carbon sources have a significant influence on microbial community structure (Bienhold et al., 2011; Sawall et al., 2012; Raulf et al., 2015).

## Conclusion

Coastal sandy sediments have a higher permeability and lower porosity than the silty clays that constitute deep-sea sediments, which in turn results in a lesser interstitial volume and lesser overall particle surface. These sediment properties combined with the prevalent hydrodynamics due to wave action, currents and gas ebullition create microbial habitats in shallow methane seeps that are very different from those found at methane seeps in the deep sea. To distinguish these habitats we think that a standardized and detailed ontology of methane-fuelled ecosystems is needed. Our findings suggest that the high phylogenetic and morphological diversity of anaerobic methanotrophs, and the apparently low-efficient methane filter at the Pomonte seep site are linked to the sediment characteristics of the ecosystem. Yet, the underlying environmental processes that shape microbial diversity, abundance and function remain unclear and are promising objectives of further research. The study underlines that our understanding of shallow-water methane seeps is still incomplete, despite their widespread occurrence on active and passive continental margins and importance for the global methane budget. It is crucial to further investigate the microbial ecology and efficiency of methane removal as most of the emitted methane at shallow seeps is released directly to the atmosphere.

## Author Contributions

All authors were involved in the design and the writing of the study. CL, MW, HK, and JW sampled in the field. HK, JW, and GW processed the samples in the lab. ER, HK, GW, AR, JW analyzed the data.

## Conflict of Interest Statement

The authors declare that the research was conducted in the absence of any commercial or financial relationships that could be construed as a potential conflict of interest.
