# Supplementary material for: Methane Seep in Shallow-Water Permeable Sediment Harbors High Diversity of Anaerobic Methanotrophic Communities, Elba, Italy
Source: Front Microbiol. 2016 Mar 31;7:374. doi: 10.3389/fmicb.2016.00374 (PMC4814501; doi:10.3389/fmicb.2016.00374)
Supplement: Supplementary file 1 [file Image_1.PDF]

Supplementing Material

Ruff SE, Kuhfuss H, Wegener G, Lott C, Ramette A, Wiedling J, Knittel K and Weber M (2016) Methane Seep in Shallow-Water Permeable Sediment Harbors High Diversity of Anaerobic Methanotrophic Communities, Elba, Italy. Front. Microbiol. 7:374. doi: 10.3389/fmicb.2016.00374

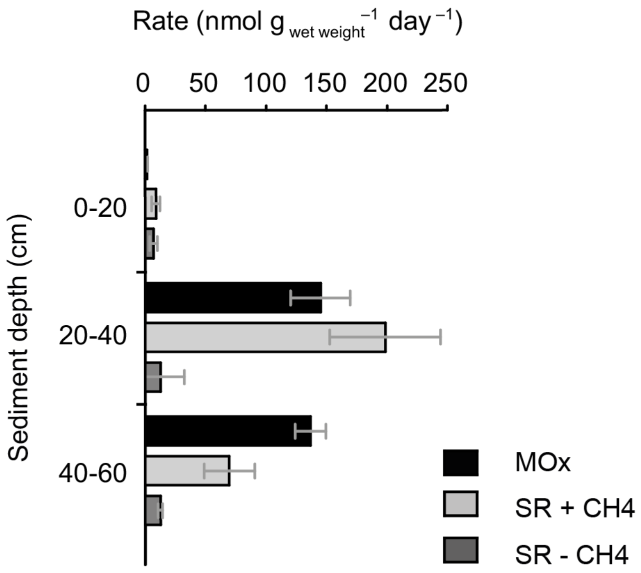

Figure S1: Rates of methane oxidation (MOx) and sulfate reduction (SR) in three different depth horizons at emission spot 1. Sulfate reduction was measured with (+) and without (-) the presence of methane (CH_4). Bars depict standard error.

A

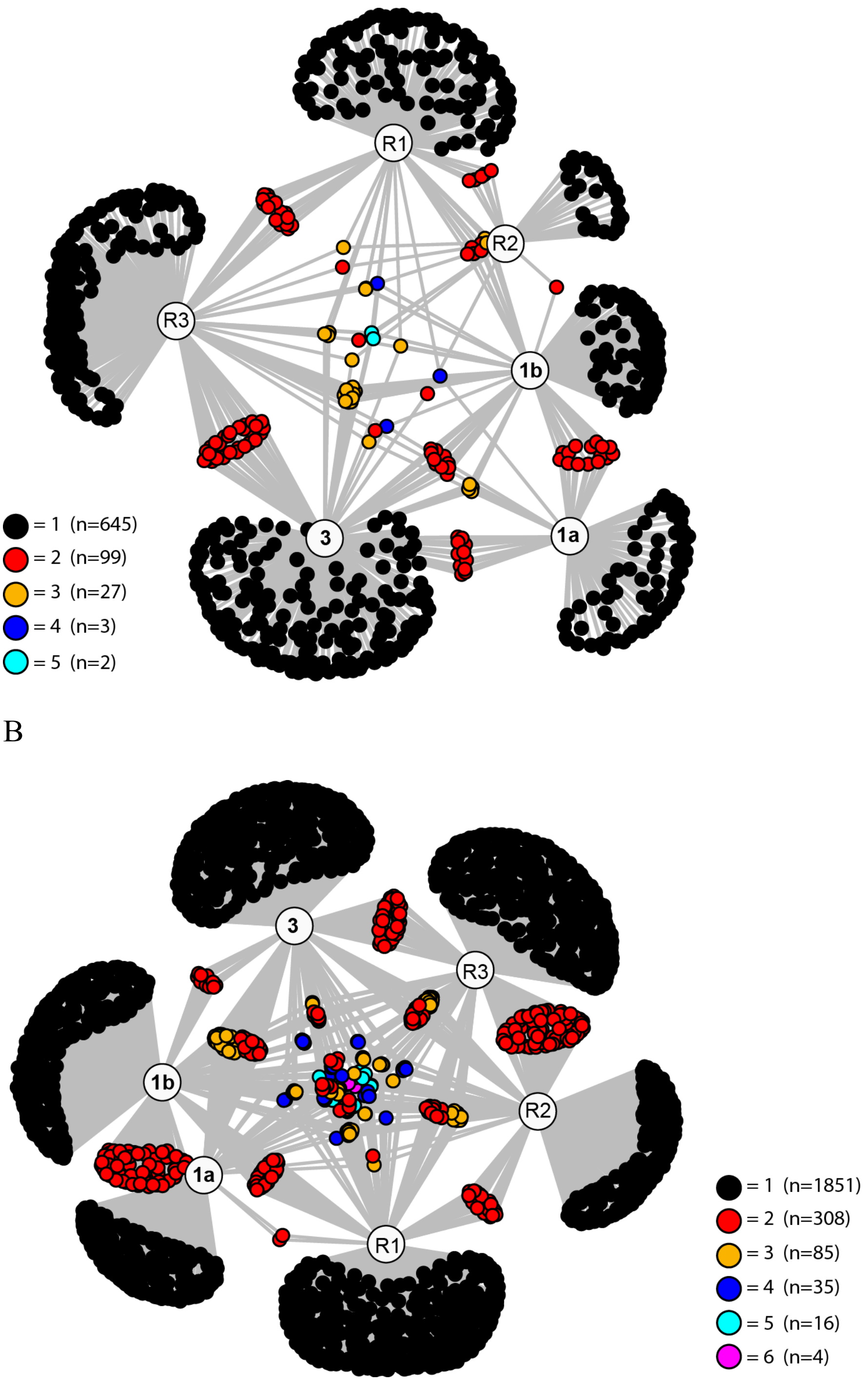

Figure S2: Network graph depicting the connectivity of sediment samples (ES1a, ES1b, ES3, Ref1-3) based on archaeal (A) and bacterial (B) operational taxonomic units at 98% identity of the 16S rRNA gene V3-V5 region ($OTU_{0.02}$). Each node (circle) represents an individual $OTU_{0.02}$. Grey lines connect an OTU to the sample (white large circles) at which it was found. The colour of the node indicates the number of samples in which this OTU was found, e.g. black dots represent OTUs that were only found at one emission spot, while pink OTUs were found in all six sediments.

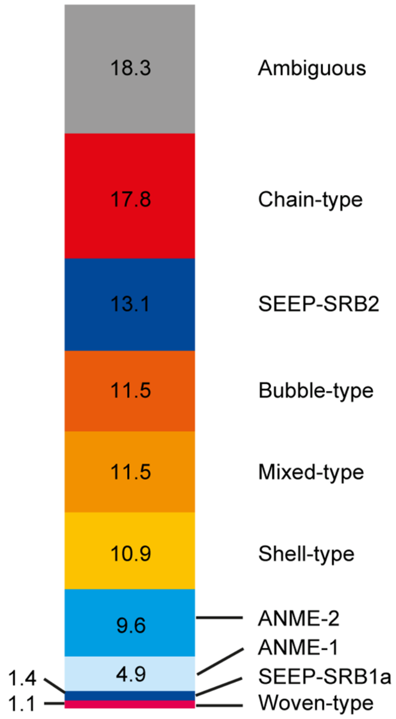

Figure S3: Relative abundance (in %) of aggregate types detected in the sediment of the emission spots 1, 3, and 4. The total number of visualized and evaluated aggregates was 366. Multispecies aggregates (bubble-, chain-, mixed-, shell-, woven-type morphology) are shown in orange colors, monospecies aggregates are shown in blue. Aggregates that did not clearly belong to one of the morphology types are marked as ambiguous.

Table S1: List of the investigated emission and reference spots at the Pomonte methane seep site off the coast of the Tuscan Island Elba in the Northern Tyrrhenian Sea (Italy).

	**Sampling Date**	**Water temperature**	**Currents**	**Weather Conditions**	**Sampled depths (cm bsf)**
Emission spot 1a	15.10.2009	19°C	medium to strong	Calm and stable	0-10
					30-40
					50-60
Emission spot 1b	07.05.2010	12°C	medium	Stormy and heavy rains	0-10
					10-20
					20-30
Emission spot 3	07.05.2010	12°C	medium		0-10
					10-20
					20-30
					30-40
Emission spot 4	07.05.2010	12°C	medium		0-10
					10-20
					20-30
					30-40
Reference 1-3	26.10.2012	19°C	medium	Calm and stable	0-10
					10-20
					20-30
					30-40
					40-50

Table S2: Horseradish-peroxidase labelled oligonucleotide probes used in this study.

Oligo-nucleotides	Specificity	Position	FA (% v/v)	Nucleotide Sequence 5'-3'	Reference
ANME-1-350	ANME-1	350-367	40	AGT TTT CGC GCC TGA TGC	(Boetius et al., 2000)
ANME-2-538	ANME-2	538-555	50	GGC TAC CAC TCG GGC CGC	(Treude et al., 2005)
ANME-2a-647	ANME-2a	647-664	35	TCT TCC GGT CCC AAG CCT	(Knittel et al., 2005)
ANME-2c-760	ANME-2c	760-777	50	CGC CCC CAG CTT TCG TCC	(Knittel et al., 2005)
DSS658	*Desulfosarcina/ Desulfococcus*	658-675	50	TCC ACT TCC CTC TCC CAT	(Manz et al., 1992)
Eub338-I	most*Bacteria*	338-355	35	GCT GCC TCC CGT AGG AGT	(Amann et al., 1990)
Eub338-II	most*Planctomycetales*	338-355	35	GCA GCC ACC CGT AGG TGT	(Daims et al., 1999)
Eub338-III	*mostVerrucomicrobiales*	338-355	35	GCT GCC ACC CGT AGG TGT	(Daims et al., 1999)
NON338	Nonsense probe	-	35	ACT CCT ACG GGA GGC AGC	(Wallner et al., 1993)
SEEP1a-1441	SEEP-SRB-1a	1441-1458	45	CCC CTT GCG GGT TGG TCC	(Schreiber et al., 2010)
SEEP-2-658	SEEP-SRB-2	658-675	45	TCC ACT TCC CTC TCC GGT	(Kleindienst et al., 2012)

Table S3: Porewater profiles of emission spots 1, 2, 3 (ES1-3) and a reference spot. N.a. = not available.

Emission Spot*	depth (cm)	Methane (mM)	DIC (mM)	δ13C-DIC (‰ vs PDB)	Alkalinity (mM)	Sulfate (mM)	Sulfide (mM)
**ES1**	**50**	n.a.	2.6	-2.05	2.7	29.0	0.00
	0	0.00	2.6	-2.08	2.7	28.6	0.00
	-10	0.10	2.8	-2.47	2.7	28.9	0.00
	-20	0.55	2.7	-5.38	3.6	30.8	0.17
	-30	0.12	2	-4.79	4.6	29.3	0.66
	-40	0.00	3.1	-2.6	4.6	31.3	0.78
	-50	0.00	3.4	-4.01	5.3	29.0	1.11
	-60	n.a.	4.6	-6.4	8.9	25.9	1.78
**ES2**	50	n.a.	3.1	-1.91	2.3	30.3	0.00
	0	n.a.	3	-2.1	2.5	28.4	0.00
	-10	n.a.	9	-10.13	13.7	20.6	7.84
	-20	n.a.	8.3	-9.43	17.1	23.0	8.97
	-30	n.a.	6.3	-8.92	15.2	22.6	7.17
	-40	n.a.	7.4	-8.15	14.3	23.7	7.09
	-50	n.a.	7.8	-8.53	12.9	23.7	5.38
**ES3**	50	n.a.	3.2	-1.5	2.5	28.5	0.00
	0	0.00	3.2	-1.78	2.4	30.0	0.00
	-10	0.05	5.3	-1.97	6	31.0	2.14
	-20	0.12	4	-6.79	5.1	30.2	1.19
	-30	0.17	3.8	-3.11	4.9	30.2	1.10
	-40	0.04	3.8	-3.73	4.4	32.1	0.84
	-50	n.a.	3.9	-3.91	4.1	28.4	0.80
	-60	n.a.	3.8	-3.56	4.5	29.9	1.01
**Reference**	50	n.a.	2.6	-1.91	2.4	28.5	0.00
	0	0.00	2.6	-1.75	2.4	30.6	0.00
	-10	0.00	2.6	-1.75	2.4	29.9	0.00
	-20	0.00	2.6	-1.93	3.2	32.3	0.00
	-30	n.a.	2.6	-1.87	2.9	32.0	0.00
	-40	0.00	2.6	-1.93	3.2	30.7	0.00
	-50	n.a.	2.7	-2.03	2.7	29.1	0.00
	-60	n.a.	1.7		2.7		

* Each emission spot was sampled once and no technical replicates were measured.

Table S4: Bray-Curtis and Jaccard distances between samples based on archaeal and bacterial $OTU_{0.02}$.

		ES1a	ES1b	ES3	Ref1	Ref2	Average All	Average ES vs ES	Average Ref vs Ref	Average ES vs Ref
**Bray-Curtis**	**ES1b**	0.68					0.79	0.70	0.58	0.89
**Distance**	**ES3**	0.57	0.84							
**Archaea**	**Ref1**	1.00	0.69	0.99						
	**Ref2**	1.00	0.71	0.98	0.28					
	**Ref3**	0.99	0.75	0.91	0.72	0.75				
**Bray-Curtis**	**ES1b**	0.73					0.86	0.79	0.76	0.91
**Distance**	**ES3**	0.78	0.84							
**Bacteria**	**Ref1**	0.96	0.88	0.93						
	**Ref2**	0.95	0.92	0.88	0.81					
	**Ref3**	0.95	0.92	0.84	0.81	0.66				
**Jaccard**	**ES1b**	0.81					0.87	0.82	0.71	0.94
**Distance**	**ES3**	0.73	0.92							
**Archaea**	**Ref1**	1.00	0.82	0.99						
	**Ref2**	1.00	0.83	0.99	0.44					
	**Ref3**	1.00	0.86	0.95	0.84	0.85				
**Jaccard**	**ES1b**	0.84					0.92	0.88	0.86	0.95
**Distance**	**ES3**	0.88	0.91							
**Bacteria**	**Ref1**	0.98	0.94	0.96						
	**Ref2**	0.97	0.96	0.93	0.895					
	**Ref3**	0.97	0.96	0.92	0.897	0.79				

References

Amann, R. I., Binder, B. J., Olson, R. J., Chisholm, S. W., Devereux, R. and Stahl, D. A. (1990). Combination of 16S rRNA-targeted oligonucleotide probes with flow cytometry for analyzing mixed microbial populations. *Appl. Environ. Microbiol.* 56**,** 1919-1925.

Boetius, A., Ravenschlag, K., Schubert, C. J., Rickert, D., Widdel, F., Gieseke, A., et al. (2000). A marine microbial consortium apparently mediating anaerobic oxidation of methane. *Nature* 407**,** 623-626.

Daims, H., Brühl, A., Amann, R., Schleifer, K. H. and Wagner, M. (1999). The domain-specific probe EUB338 is insufficient for the detection of all Bacteria: development and evaluation of a more comprehensive probe set. *Syst. Appl. Microbiol.* 22**,** 434-444.

Kleindienst, S., Ramette, A., Amann, R. and Knittel, K. (2012). Distribution and in situ abundance of sulfate-reducing bacteria in diverse marine hydrocarbon seep sediments. *Environ. Microbiol.* 14**,** 2689-2710.

Knittel, K., Lösekann, T., Boetius, A., Kort, R. and Amann, R. (2005). Diversity and Distribution of Methanotrophic Archaea at Cold Seeps. *Appl. Environ. Microbiol.* 71**,** 467-479.

Manz, W., Amann, R., Ludwig, W., Wagner, M. and Schleifer, K.-H. (1992). Phylogenetic Oligodeoxynucleotide Probes for the Major Subclasses of Proteobacteria: Problems and Solutions. *Syst. Appl. Microbiol.* 15**,** 593-600.

Schreiber, L., Holler, T., Knittel, K., Meyerdierks, A. and Amann, R. (2010). Identification of the dominant sulfate-reducing bacterial partner of anaerobic methanotrophs of the ANME-2 clade. *Environ. Microbiol.* 12**,** 2327-2340.

Treude, T., Knittel, K., Blumenberg, M., Seifert, R. and Boetius, A. (2005). Subsurface Microbial Methanotrophic Mats in the Black Sea. *Appl. Environ. Microbiol.* 71**,** 6375-6378.

Wallner, G., Amann, R. and Beisker, W. (1993). Optimizing fluorescent in situ hybridization with rRNA-targeted oligonucleotide probes for flow cytometric identification of microorganisms. *Cytometry* 14**,** 136-143.
